# Barriers, facilitators, needs, and preferences in seeking information regarding cervical cancer prevention programs among Turkish, Moroccan, and Syrian immigrant women: a scoping review

**DOI:** 10.1186/s12889-025-22359-2

**Published:** 2025-04-02

**Authors:** Tharsini Veeravagu, Nora Hamdiui, Mart L. Stein, Rik Crutzen, Aura Timen

**Affiliations:** 1 National Coordination Centre for Communicable Disease Control, Centre for Infectious Disease Control, National Institute for Public Health and the Environment, Bilthoven, The Netherlands; 2 Radboud University Medical Center, Radboud Institute for Health Sciences, Department of Primary and Community Care, Nijmegen, The Netherlands; 3 Department of Health Promotion, Care and Public Health Research Institute, Maastricht University, Maastricht, The Netherlands

**Keywords:** Cervical cancer screening, HPV vaccination, Informed decision-making, Immigrant women, Information needs

## Abstract

**Background:**

Cervical cancer (CC) is the fourth most frequently diagnosed cancer in women worldwide. Immigrant women are often disproportionately affected by CC but show low participation in CC screening and human papillomavirus (HPV) vaccination.

**Methods:**

We conducted a scoping review on immigrant women’s information needs regarding CC screening participation and HPV vaccination uptake. A total of 584 articles were found on Embase.com, PsychINFO, and CINAHL, of which 87 articles were included.

**Results:**

This review revealed that immigrant women indicate a need for more personalized information regarding CC screening and HPV vaccination. We identified barriers to obtaining, processing, and understanding the information, which included overall practical, emotional, cultural and religious aspects (e.g., shame, taboo, lack of trust, fatalism, and cultural norms and values regarding sexual activity). Facilitators, such as translation services, receiving information from people with similar cultural and/or religious backgrounds, encouraging other women or family, and using home visits as an outreach strategy, were also identified.

**Conclusions:**

Our review provides a comprehensive overview of the information needs and preferences of immigrant women, which could be used to tailor interventions, considering the contextual nuances in which these women are situated. The needs and preferences of immigrant women should be taken into account during the development of new information materials or other interventions. This would help immigrant women make informed decisions regarding participation in CC screening and HPV vaccination.

**Supplementary Information:**

The online version contains supplementary material available at 10.1186/s12889-025-22359-2.

## Background

Cervical cancer (CC) is the fourth most common cancer in women worldwide [1]. Promising efforts have been made in CC prevention programs, such as guidelines for CC screening. In 2022, 139 out of 202 (69%) countries had official CC screening recommendations [2]. In June 2020, 107 out of 194 (55%) of the World Health Organization (WHO) member states had implemented Human Papilloma Virus (HPV) vaccination [3]. In the Netherlands, since 1996, women between 30 and 60 years old have received an invitation for participation in CC screening every five years [4]. HPV vaccination was implemented in the Netherlands in 2010, but only for 12-year-old girls. Later, in 2022, they started inviting boys and girls for HPV vaccination in the year they turn 10 years old [5]. Despite the successful implementation of these programs, disparities in participation among individuals with a non-Dutch country of origin (representing 15% of the Dutch population [6]) persist in the Netherlands [7, 8]. Similar disparities were found in a study on ethnic differences in HPV awareness and vaccine acceptability in the United Kingdom among Indian, Pakistani, Bangladeshi, Caribbean, African, and Chinese women [9]. This study indicated that ethnicity and religion were strongly associated with vaccine acceptability. Fewer vaccine ‘acceptors’ were found among Muslims (18%) compared to Hindus (34%) and those with no religion (64%). The most common reasons mentioned for declining were lack of information, sexual related aspects, religious and cultural reasons, and lack of trust in terms of safety [9]. In the Netherlands, two of the largest groups of immigrants originate from Turkey and Morocco: 431 and 419 thousand individuals, respectively, in 2022 (with a population of 17.6 million individuals) [6]. These numbers include both first- and second-generation immigrants. First-generation immigrants were born in their country of origin and later migrated to the Netherlands, whereas second-generation immigrants were born in the Netherlands but had at least one parent born in their country of origin. Compared with native Dutch women, higher incidence rates of CC are reported for Turkish- and Moroccan-Dutch women [7, 8]. Additionally, Turkish- and Moroccan-Dutch women also participate less often in CC screening and exhibit lower uptake of HPV vaccination for their children [8].

A lack of good command of the Dutch language was one of the perceived barriers to CC screening participation among Turkish- and Moroccan-Dutch women [10]. Fatalism, shame, taboo, and associations of CC with a lack of femininity and infertility also pose a barrier for these women. On the other hand, having a female general practitioner could function as a facilitator for participation, as well as perceiving greater severity of the disease, social support and a short procedure time [10]. Additionally, a religious facilitator included the responsibility to take care of one’s health while making use of the medical options that are provided (by God) [10]. The needs and preferences for information on HPV vaccination have not yet been investigated among Turkish- and Moroccan-Dutch women but are expected to be in line with those found for CC screening. This is due to overlapping cultural and religious aspects regarding sexuality, monogamy, virginity, fertility, and marriage that are linked to both HPV vaccination and CC screening. Until now, the information materials for CC screening and HPV vaccination, and the way they are provided, have not been tailored to the needs and preferences of immigrant populations in the Netherlands. Information materials in different languages (i.e. English, Turkish, Arabic, Tamazight, Ukrainian, and Russian) are only available online and difficult to find. Women aged 30 to 60 years old receive an invitation letter and information brochure in Dutch, which are mainly focused on factual medical information. However, research by Hamdiui et al. [11] revealed that Turkish- and Moroccan-Dutch women have the need for information about the practical, emotional, cultural, and religious aspects of CC as well.

Besides Turkish- and Moroccan-Dutch women, another large immigrant population in the Netherlands are Syrian–Dutch women. In recent years, this group has grown enormously in size. For example, the influx in 2022 increased by 12% compared with that in the previous year [12]. Syrian women were found to have limited knowledge of HPV and HPV vaccination, while vaccine acceptability was found to be high [13, 14]. Additionally, in a previous study, Syrian refugees in Greece showed little to no awareness of CC [15]. No data on the risk of CC or CC screening participation of Syrian-Dutch women are available, but an increase in the prevalence of untreated CC cases among these women might be observed in the coming years due to the disruption of the healthcare provision in their country of origin. 

A study by Andermann revisited the criteria for offering and communicating screening programs and showed that Western medicine, including Dutch policy, focuses more on informed decision-making [16]. According to the rational decision model, informed decision-making entitles individuals to base their decisions on making maximum use of information and rationally weighing all aspects (both pros and cons) involved [17]. Deciding to participate in screening involves careful consideration of uncertain benefits (e.g., a longer duration of life if a precursor of cancer is successfully detected and treated) and the risk of adverse effects (e.g., false-positive and false-negative test results, overdiagnosis and treatment, and discomfort or pain). In the Netherlands, informed decision-making regarding CC screening participation is lacking among the general Dutch population [18]. This also persists among Turkish- and Moroccan-Dutch women because approximately half of them do not make informed decisions about their CC screening participation [10]. The recommendations for HPV vaccination are slightly different. In the United States (US), the Advisory Committee on Immunization Practices (ACIP) and the Centers for Disease Control and Prevention (CDC) recommended shared clinical decision-making (SCDM) regarding HPV vaccination. This refers to a considered decision of the individual and their healthcare provider(s), in which the latter actively encourage(s) toward taking the vaccination [19].

Previous studies have investigated the barriers to and facilitators of participation in CC screening and HPV vaccination. For example, a review conducted among different subgroups in the U.S. investigated barriers and potential solutions for participation in CC screening [20]. These barriers consisted of difficulties in interacting with the healthcare system due to limited knowledge of CC and its prevention programs, insufficient health literacy, and a lack of recommendations by healthcare professionals. Additionally, financial and logistical barriers were identified (e.g., lack of usual sources of care, scheduling issues). Another review identified four themes relevant to decision-making concerning HPV vaccination uptake: (1) Fear and risk of the vaccine and the disease, (2) Pain from receiving the vaccine, (3) Parental involvement, and (4) Involvement of others [21]. However, an exploration of how immigrant women obtain, process, and understand information regarding CC, CC screening, and HPV vaccination is lacking. Therefore, this scoping review is guided by the following research questions:

1) What are the current knowledge and beliefs among Turkish, Moroccan, and Syrian women regarding CC, CC screening, and HPV vaccination?

2) What are the barriers and facilitators for Turkish, Moroccan, and Syrian women in obtaining, processing, and understanding information regarding CC, CC screening, and HPV vaccination?

3) What are the needs and preferences of Turkish, Moroccan, and Syrian women in obtaining, processing, and understanding information regarding CC, CC screening, and HPV vaccination?

## Methods

This study applied a scoping review methodology, which is a comprehensive approach facilitating the systematic exploration and mapping of literature. The scoping review is presented according to guidelines in the PRISMA statement 2020 (see Appendix A, Table A1 for the checklist) [22].

### Data collection

To identify relevant articles, we searched Embase.com (including PubMed), PsycINFO, and CINAHL. The search strings used in each database contained keywords such as ‘prevention and control’, ‘health education, ‘vulnerable population’, ‘migrant’, ‘uterine cervix tumor’, and ‘human papilloma virus vaccine’ (the complete search strings are provided in Appendix B, Tables B1, B2, and B3). The search in Embase.com and PsycINFO was performed in February 2023 and updated in April 2024. In January 2025, we conducted a search in the CINAHL database which resulted in 13 additional relevant articles. Duplicates were removed before article selection took place.

### Article selection

The articles were independently assessed by two researchers (TV and NH), who considered the inclusion criteria (described below under ‘Inclusion criteria’). This was done by screening the titles and abstracts for their eligibility and relevance to the study population, concept, and context (described under ‘Inclusion criteria’). The online platform Rayyan was used to identify discrepancies in the inclusion and exclusion of articles between the two researchers. These were discussed until a consensus was reached on whether to include an article. A full-text analysis (described in detail under ‘Data analysis’) was subsequently conducted.

### Inclusion criteria

Article selection was conducted on the basis of the inclusion criteria presented in Table 1. These criteria were tailored to the research questions of this study and based on the population/concept/context (PCC) framework [23]. We included immigrant populations with a comparable culture and/or religion, such as norms and values regarding sexual activity and marriage. For example, studies among immigrants from Somalia, Egypt, Eritrea, Pakistan, and other countries with Islam as a prominent religion were included. Furthermore, we also included studies conducted within such countries. Studies that focused only on the barriers to and facilitators of participation in CC screening and HPV vaccination and did not further investigate the points described under ‘Concept’ (see Table 1) were excluded from this review. We only included studies focusing on the general population from Turkish, Moroccan, and/or Syrian origin (or a comparable country of origin in terms of culture and religion). This means that studies focusing on specific subgroups, such as ‘students’ or ‘healthcare professionals’, were excluded from our review.

**Table 1:** Inclusion criteria

Inclusion criteria
**Population:** • First- and second-generation immigrants (women) from Turkey, Morocco, and/or Syria (or a comparable country of origin in terms of culture and religion) • General population from Turkish, Moroccan, and/or Syrian origin (or a comparable country of origin in terms of culture and religion), (no specific subgroups such as ‘students’ or ‘healthcare professionals’)
**Concept** • Information seeking behavior regarding CC screening and/or HPV vaccination • Perceived barriers and facilitators in obtaining, processing and understanding information regarding CC screening and/or HPV vaccination • Needs and preferences for information about CC screening and/or HPV vaccination
**Context** • Immigrants living in high income countries • Individuals living in Turkey, Morocco, or Syria (or countries with similarities in terms of culture and religion)
**Other** • Written in English • Published in a peer-reviewed journal

### Data analysis

To conduct the full-text data analysis, a data extraction sheet was developed beforehand and used to collect the relevant information from the included articles. The extraction of data was driven by and based on the research questions and inclusion criteria of this study. This resulted in the following data categories: ‘first author’, ‘year published’, ‘country’, ‘aim’, ‘relevance’, ‘type of preventative care’, ‘method’, ‘theoretical background’, ‘study design’, ‘study population’, ‘recruitment’, ‘information resources’, ‘knowledge and perceptions’, ‘misconceptions’, ‘barriers and facilitators for obtaining, processing, and understanding the information’, ‘norms and beliefs’, ‘cultural aspects’, and ‘information needs and preferences’. During full-text analysis, the data extraction sheet was filled in for all of the included articles by one researcher (TV). The use of certain theoretical frameworks was not considered as inclusion or exclusion criteria. However, we did extract them to provide a quantitative representation of theoretical frameworks in the included studies in this review. The quality appraisal checklist developed by the Critical Appraisal Skills Programme (CASP) was also integrated to assess the quality of the articles [24]. Thereafter, a second researcher (NH) reviewed the data extraction sheet. Discrepancies in including and excluding articles between the two researchers were discussed until consensus on whether to include articles was reached.

## Results

### Search results

**Fig. 1:**
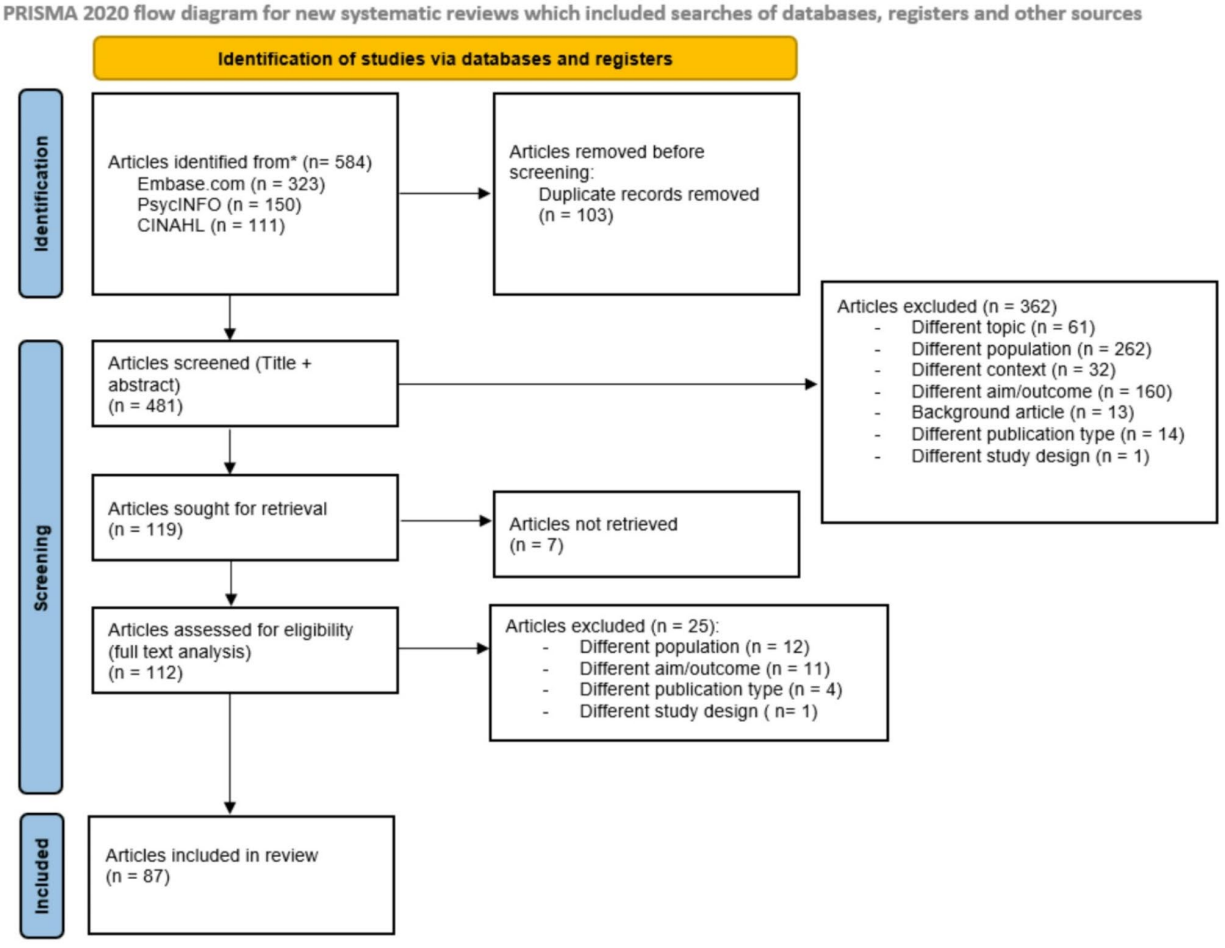
Prisma 2020 diagram for new systematic reviews

The Prisma flowchart presents the study selection procedure (see Fig. 1). In total, 584 articles were derived from the three databases. After correcting for duplicates, 481 articles were screened on the basis of title and abstract. This resulted in 362 articles being excluded because of different topics, populations, contexts, or other reasons (i.e., articles focused only on participation in CC screening and HPV vaccination, different publication types (master’s/PhD theses), or different study designs). The remaining 119 articles were searched for full-text versions, of which 7 articles were not retrievable due to a lack of open access and/or limited access rights, and there was no response after the original authors were contacted. In total, 112 articles were included in the full-text screening. Throughout this process, another 25 articles were excluded because of different study populations, study objectives, publication types, and study design. This scoping review included 87 original articles from which data were extracted.

### Study characteristics

Table 2 shows an overview of the articles included in this review (*n* = 87). Most studies were conducted in the US (*n* = 19) or Turkey (*n* = 24). All the study samples consisted of women who were eligible for CC screening or parents having children eligible for HPV vaccination. In total, 34 articles had a qualitative study design (e.g., interviews and focus group discussions), 42 were quantitative studies (e.g., questionnaires ), and 11 mixed-methods studies were found. The majority of the included articles did not use a theoretical background for their study (*n* = 41). Of those that did, most used the Health Belief Model (HBM) (*n* = 23), followed by the Theory of Planned Behavior (*n* = 3). Most of the included articles focused on CC screening (*n* = 41). Five of these articles also described factors related to breast cancer or colorectal cancer. HPV vaccination was described in 34 articles, and 12 articles focused on both CC screening and HPV vaccination. The aim of the studies varied from investigating the barriers, facilitators and the intention to participate in CC screening and HPV vaccination to experiences, knowledge, and awareness of these programs. Overall, 24 of the included studies focused on the provision of information and the decision-making process regarding participation in CC screening and HPV vaccination. Furthermore, all included studies scored at least 4 out of 7 in the quality appraisal checklist [24] (Table 2).

**Table 2:** Description of the included articles

Author (year)	Context	Aim	Study design	Study population (*n*)	Quality appraisal score					
U. Abakporo (2018)	USA	To inform future behavioral interventions in increasing uptake of HPV vaccination and CC screening among Somali adolescents and women, and to generate hypotheses for future generalizable research into the topic.	One-on-one interviews	Married Somali men, aged 25–55, with children (*n* = 30)	7					
A. Adegboyega (2023)	USA	To explore HPV beliefs, attitudes, and knowledge among Black young adults with a view of providing recommendations on strategies to improve HPV vaccine uptake among Black young adults.	Focus group discussions and questionnaire	Black young adults (*n* = 38)	6					
E. Agadayi (2022)	Turkey	To investigate HPV knowledge and behaviors of women who have or have not had HPV vaccine.	Questionnaire	Women between 18–65 year (*n* = 856)	6					
M.B. Akbas (2023)	Turkey	To evaluate the relationship between health literacy levels and HPV vaccination attitudes of parents.	Questionnaire	Parents with children between 0–18 or persons aged 18+ (*n* = 418)	6					
G. Akca (2022)	Turkey	To evaluate the knowledge and attitude of Turkish mothers about cervix cancer, HPV infection and vaccination.	Questionnaire	Mothers with children between 0–18 (*n* = 330)	6					
O.M. Akhagba (2017)	Poland	To explore the knowledge and perceptions of African women about CC and its screening programs available in Poland.	Focus group discussions	Women between 25–54 from Egypt, Eritrea, Kenya and Nigeria (*n* = 12)	4					
S. Al Alawi (2023)	Oman	To assess knowledge, attitudes, and acceptance toward HPV vaccination in men and women from Oman, a country in the Middle Eastern North Africa region.	Questionnaire	Omani residents (*n* = 1403)	6					
C.J. Alberts (2017)	The Netherlands	To explore possible impact of ethnicity on the determinants of both HPV-vaccination intention and uptake among parents/guardians having a daughter that is invited.	Questionnaire	Parents/guardians with a daughter born in 2001, living in the district of the Youth Health Service of the Public Health Service of Amsterdam (*n* = 1309)	7					
J.A. Anaman (2017)	Australia	To compare the level of CC screening uptake between refugee and non-refugee African immigrant women living in Brisbane, Australia, and examine factors associated with Pap smear testing.	Questionnaire	African-born women, aged 21–65, living in Brisbane (*n* = 254)	5					
Z. Arabaci (2012)	Turkey	To examine the attitude of women who have pap-smear test for the early diagnosis of cervical cancer, factors affecting their decisions and their feelings and experiences during this period.	One-on-one interviews	Women, 35 years and older, married/sexually active, at least one pap-smear (*n* = 17)	6					
C. Ayash (2022)	USA	To explore associations between US Arab immigrant mothers’ beliefs regarding HPVV for their children with socioeconomic, medical, and religious/cultural factors.	Questionnaire	Arab immigrant women aged 21–65, with at elast one child aged 9–26. Quantitative data (*n* = 162), qualitative data (*n* = 100)	7					
S. Badre-Esfahani (2021)	Denmark	To explore perceptions of and barriers to HPVV and CCS, among MENA and Pakistani women in Denmark.	Focus group discussions	Immigrant or descendants with parents from MENA and Pakistan, aged 23–65, and fluently speaking Danish (*n* = 17)	6					
I.Basnyat (2017)	Singapore	To examine the means by which young Singaporean women seek and process information about HPV vaccination in their decision to become vaccinated.	One-on-one interviews	Chinese Singaporean women aged 18–26 (*n* = 26)	6					
H. Batista Ferrer (2016)	England	To identify the barriers and facilitators to uptake of the HPV vaccine in an ethnically diverse group of young women in the South west of England.	One-on-one interviews and observations (during vaccination session)	Key informant interviews with school staff (*n* = 3) and nurses (*n* = 3), and young women black/black British/Asian, Asian/white and other/mixed) (*n* = 23)	7					
N.Ç. Bilgin (2022)	Turkey	To investigate the effect of group education on human papillomavirus (HPV) on level of knowledge and health beliefs for HPV infection and vaccine.	Questionnaire	Mothers of secondary schools (*n* = 110)	6					
I.R. Bou-Orm (2018)	Lebanon	To assess the prevalence of Pap smear screening for cervical cancer among Lebanese women and to determine associated sociodemographic and psychosocial characteristics.	Questionnaire	Women between 18–65 years in Lebanon, without Gynecological history of infections (*n* = 2255)	5					
P. Çelik (2021)	Turkey	To determine parents awareness, knowledge, attitude and perceptions about HPV infection and HPV vaccine in Turkey.	Questionnaire	Parents with children aged 9–18 years (*n* = 998)	6					
D.N.S. Chan (2017)	Worldwide	To examine the factors that influence ethnic minority women in using CC screening and the similarities and differences in associated factors across different groups and to explore the interrelationships between the factors identified.	Literature review	Studies exploring factors influencing women’s CC screening behavior among ethnic minority women (*n* = 23)	5					
D.N.S. Chan (2022)	Hong Kong	To examine the feasibility and acceptability of a linguistically appropriate printed decision aid for cervical cancer screening in South Asian women and to preliminarily estimate its effects on decisional conflicts, clarity of values, risk perception, the screening decision and screening uptake.	Pilot RCT	South-Asian women (India, Pakistan, Nepal), aged 25–64 (*n* = 48)	7					
J. Cudjoe (2021)	USA	To explore how various sources and types of health information influence information sharing and health literacy in the context of cervical cancer screening among African immigrant women.	Questionnaire and one-on-one interviews	African immigrant women (survey: *n* = 167, and interviews: *n* = 20)	6					
P.M. Dailey (2017)	USA	To explore how Somali immigrant families living in Ohia, USA, make decisions regarding whether to vaccinate their children against HPV.	One-on-one interviews	Somali parents with children aged 9–17 (*n* = 20)	7					
E. Cosar (2014)	Turkey	HPV and HPV vaccination: Knowledge and consciousness of young women.	Questionnaire	Students and young women (*n* = 650)	6					
B. Demirtas (2013)	Turkey	To identify the relationship between HBM scale for CC and the Pap smear test subscale scores and demographic/gynaeco-obstetric characteristics.	Questionnaire	Turkish women, 21+, sexually active (*n* = 256)	4					
E.T. Duran (2011)	Turkey	To examine women’s attitude to the health belief model, and their attitudes and behavior towards CC and early diagnosis.	Questionnaire and One-on-one interviews	Women between 15–49, married and no previous Pap-smear test (*n* = 11)	6					
N.I.E. Enyan (2022)	Ghana	To investigate Muslim women’s participation in, attention to, engage in, and self-efficacy about cervical cancer screening.	Questionnaire	Muslim women aged 18–66 (*n* = 431)	7					
R. Erenoğlu (2020)	Turkey	To evaluate the effect of health education given to refugee women in their own language on the awareness of breast and cervical cancer.	Questionnaire	Syrian refugee women (*n* = 60)	7					
M.N. Esin (2011)	Turkey	To determine the beliefs of women about CC and the influencing factors.	Questionnaire	Married Turkish women, aged 16–79 (*n* = 300)	7					
H. Fisher (2024)	England	To understand the information needs of vaccine-hesitant, ethnically diverse parents during decision-making about the HPV vaccine for their adolescent child, to inform the future development of tailored communication materials.	One-on-one interviews	Parents who refused consent or had not responded to an invitation (*n* = 29)	7					
S. Ford (2014)	USA	To examine fidelity and consistency of treatment delivery and assess qualitative elements of the intervention.	Questionnaire	Immigrant women (*n* = 305) and CHW (*n* = 16)	5					
Y. Gendler (2024)	Israel	To assess the impact of a novel Web-based decision aid on HPV vaccination intentions, knowledge, decision self-efficacy, and decisional conflict among Israeli parents and young adults, with a specific focus on exploring differences between religious groups.	Questionnaire	Parents of children aged 10–17 (*n* = 120) and young adults (*n* = 160)	6					
B.A. Glenn (2015)	USA	To understand demographic factors associated with HPV awareness among low-income, ethnic minority mothers in LA county.	Questionnaire	Mothers of low-income ethnic minority adolescent girls (*n* = 490)	5					
D. Graci (2024)	Worldwide	To evaluate barriers to and facilitators for accessing HPV vaccination in immigrant and refugee populations.	Systematic review	Studies on international immigrants, refugees, asylum seekers, regular immigrants, immigrants in irregular situations, economic immigrants, and internally displaced persons (*n* = 34)	5					
M. Grandahl (2015)	Sweden	To explore immigrant women’s experiences and views on the prevention of cervical cancer, screening, HPV vaccination and condom use.	Focus group discussions	Immigrant women (*n* = 50)	5					
G. Gulten (2012)	Turkey	To determine the breast, cervical, and colorectal cancer screening rates and the influencing factors in a group of Turkish females.	Questionnaire	Turkish women 30+ (*n* = 603)	7					
A.G. Guven (2023)	Turkey	To determine parents’ current attitudes and beliefs using a standardized scale towards Human Papilloma Virus and its vaccine during COVID-19 Pandemic.	Questionnaire	Parents with a daughter aged 9–16 (*n* = 303)	6					
G. Guvenc (2013)	Turkey	To determine the effect of a three-stage nursing intervention to increase Turkish women’s participation in pap-smear testing.	One-on-one interviews	Turkish women, 21+ (*n* = 2500)	6					
N. Hamdiui (2022)	The Netherlands	To develop a short culturally sensitive educational video to facilitate informed decision-making regarding CC screening participation.	Developing culturally sensitive educational videos	Not applicable	6					
N. Hamdiui (2022)	The Netherlands	To evaluate the added effect of the CSEV on IDM regarding CC screening participation among Turkish and Moroccan women aged 30 to 60 years in the Netherlands through a randomized intervention study.	Questionnaire	Turkish- and Moroccan-Dutch women aged 30 to 60 years (*n* = 1564)	7					
N. Hamdiui (2021)	The Netherlands	To explore how and why Turkish- and Moroccan-Dutch women decide to participate or not in the current Dutch CC screening program, as well as to learn their perceptions on self-sampling.	Focus group discussions	First- and Second generations Turkish- and Moroccan-Dutch women aged 30–60 years old (*N* = 24 and *N* = 20 respectively)	7					
D.M. Harper (2021)	USA	To determine the rates and predictors of CC and CRC screening for women 50–65 years of three race/ethnicities.	Questionnaire	MENA Women aged 50–65 years of age (*n* = 394)	7					
F. Hilverda (2021)	The Netherlands	To explore barriers and motivators to use self-sampling kits for HPV-testing for CC screening as perceived by Dutch women of Turkish and Moroccan origin living in the Netherlands.	One-on-one interviews	Women of Turkish and Moroccan origin living in the Netherlands (*n* = 12)	7					
R. Hofman (2013)	The Netherlands	To explore decisional strategies and factors that could guide HPV-vaccination intentions.	Focus group discussions	Dutch and Turkish parents of children aged 8–15 (*n* = 36)	7					
E. Ilter (2010)	Turkey	To examine knowledge about Pap smear test, HPV, HPV vaccine and their attitude toward vaccination to themselves and their daughters.	Questionnaire	Muslim Turkish women aged 19–53 (*n* = 525)	5					
C.E. Johnson (2008)	USA	To systematically review all studies examining sociocultural factors influencing cervical cancer screening among immigrant and ethnic minorities in the United States along the theoretical framework of the Health Belief Model.	Systematic review	US-based studies (*n* = 55)	5					
V.W. Jongen (2021)	The Netherlands	To explore whether HPV-vaccination intention of the parent and that of their 12–13 year-old daughter affects actual HPV-vaccination uptake, stratified by Dutch and non-Dutch origin.	Questionnaire	Daughters aged 12–13 years and their parents (*n* = 438)	7					
O. Karabulutlu (2013)	Turkey	To determine the status of Turkish women regarding participation in Pap smear testing and affecting factors.	One-on-one interviews and questionnaire	Maried women aged 18–61 (*n* = 301)	7					
S.A. Karaoglu (2022)	Turkey	To evaluate knowledge, attitudes, and behaviors of adults about adult vaccines.	Questionnaire	Patients, 18+ (*n* = 182)	7					
A. Khan (2023)	Canada	(a) To determine current-state-of-science on the factors that influence the uptake of HPV vaccine across English Canada, (b) To explore people’s perspectives on the uptake of the HPVV through school-based programs at three levels: patients-, providers- and system-level, across Saskatchewan, and (c) To determine the COVID-19 pandemic related disruption of the school-based program HPVV program across SK.	One-on-one interviews, questionnaire and focus group discussions	Black, south Asian, and south-east Asian parents and caregivers (Interviews; *n* = 15, focus group discussions; *n* = 16)	7					
S. Khan (2015)	Dubai	To explore Muslim women’s perspectives towards cervical screening in Dubai to promote strategies for increasing its uptake, thereby leading to a decrease in morbidity and mortality associated with CC.	One-on-one interviews	Muslim women, 18+ (*n* = 13)	7					
M. Khazaee-Pool (2018)	Iran	To explore the perceptions and experiences of Iranian women regarding cervical cancer-preventive behaviors.	One-on-one interviews and focus group discussions	Women (*n* = 27)	5					
Z. Koc (2019)	Turkey	To determine the effect of education about cervical cancer and human papillomavirus on the healthy lifestyle, behavior, and beliefs of Turkish women who were without cancer, using the PRECEDE education model.	One-on-one interviews and questionnaire	Turkish women (*n* = 156)	6					
J. Lechuga (2012)	USA	To understand whether mothers from diverse ethnicities perceive a need for a decision support tool.	Questionnaire	Hispanic, non-hispanic white, and African American mothers (*n* = 150)	6					
P.W. Li (2009)	Malaysia	To assess the mother’s knowledge and attitudes toward HPV vaccination.	Focus group discussions	Malaysian mothers (*n* = 47)	6					
L.A.V. Marlow (2009)	UK	(1) To explore demographic predictors of HPV vaccine acceptability. (2) To explore HBM constructs (perceived susceptibility, perceived severity, barriers and benefits) as predictors of HPV vaccine acceptability. (3) To test the hypotheses that demographic/cultural differences in acceptability are explained by attitudinal factors.	Questionnaire	Female students, aged 16–19 (*n* = 386)	5					
L.A.V. Marlow (2015)	UK	To explore self-perceived barriers to cervical screening attendance among ethnic minority women compared to white British women.	One-on-one interviews	Women aged 25–64 years (*n* = 54)	7					
P. Marques (2021)	Portugal	To explore the perspectives of healthcare workers and community workers on the participation of migrant women in CCS in Portugal, by (i) assessing their experiences and opinions about CCS participation of immigrant women, (ii) exploring the barriers faced by these women to participate in CCS, and (iii) identifying strategies to overcome these barriers.	Focus group discussions	Healthcare workers (*n* = 12) and community workers (*n* = 5)	6					
P. Marques (2020)	Europe	To provide a synthesis of the growing evidence on factors associated with participation in CC screening among immigrant women in EU.	Scoping literature review	Studies focused on participation in CCS among immigrant women (*n* = 20)	5					
M. Matin (2004)	USA	(1) To examine the impact of religious and cultural values on health care behavior of Muslim women from immigrant backgrounds in the San Francisco Bay Area, particularly with regard to cervical cancer screening; (2) To determine whether these women would welcome discussing values and beliefs regarding sexuality and reproductive health.	Focus group discussions	Muslim women, aged 18–25 (*n* = 15)	6					
F.I. Modibbo (2016)	Nigeria	To explore the barriers to cervical cancer screening, focusing on religious and cultural factors, in order to inform group-specific interventions that may improve uptake of cervical cancer screening programs.	Focus group discussions	Women, age 18+, Christian and Muslim (*n* = 27 & *n* = 22)	7					
A.A. Mohamed (2024)	USA and Europe	To systematically review the effectiveness of interventions to improve screening adherence for breast, cervical and colorectal cancer among Somali immigrants.	Systematic literature review	Studies that evaluated interventions for cancer screening in Somali immigrant populations (*n* = 8)	5					
S.M. Mousa (2010)	USA	To find out if the intervention was effective for delivering breast and CC education.	Focus group discussions	Community health workers (*n* = 13)	6					
C. Naing (2012)	Malaysia	(1) To determine knowledge about, and perception of human papilloma virus infection in relation to cervical cancer, (2) To explore the intention of the community to be vaccinated with human papilloma virus vaccine, and (3) To identify variables that could predict the likelihood of uptake of the vaccine.	Questionnaire	Females (*n* = 232)	7					
A. Namoos (2023)	USA	To explore how cultural and religious beliefs influence the participation of Muslim women in Virginia in cervical cancer screening.	One-on-one interviews	Muslim women aged 18+ (*n* = 10)	6					
E.G. Ndukwe (2013)	USA	To investigate knowledge and awareness levels of breast and cervical cancer screening practices among female African-born immigrants to the USA residing in the Washington D.C. metropolitan area.	One-on-one interviews, focus group discussions, and questionnaire	African immigrants, aged 20–70 (*n* = 38)	5					
H.G. Öztaş (2024)	Turkey	To ascertain the impact of cervical cancer education provided to women in Turkey on their knowledge, attitudes, and health beliefs.	Questionnaire	Women (*n* = 105)	5					
N.Y. Ozturk (2021)	Australia	To describe the attitudes, beliefs, knowledge, and awareness of cervical cancer screening and screening practices among immigrant women living in Sydney, Australia.	Focus group discussions	Women from Middle Eastern, South-East Asian and African ethnicity (*n* = 52)	5					
S. Ozyer (2013)	Turkey	To assess the knowledge about HPV and HPV vaccines and attitudes towards vaccination among the females aged 9–24 years in Turkey.	Questionnaire	Turkish females, aged 9–24 (*n* = 408)	6					
A.I. Padela (2014)	USA	To assess rates of Papanicolaou (Pap) testing and associations between religion-related factors and these rates among a racially and ethnically diverse sample of American Muslim women.	Questionnaire	English speaking Muslim women (*n* = 254)	6					
R. Pratt (2019)	USA	To understand the views of Somali young adults regarding HPV immunization.	Focus group discussions	Women (*n* = 21), men (*n* = 13)	7					
R. Pratt (2020)	USA	To test the feasibility and impact of religiously tailored workshops involving Somali American Muslim women and male imams to improve intention to undergo breast or cervical cancer screening.	Questionnaire	Somali American women (*n* = 30), Imams (*n* = 11)	7					
L. Redwood-Campbell (2011)	Canada	To describe the similarities and differences among multiple groups of immigrant women and Canadian-born women of low socio-economic status regarding barriers and enablers associated with cervical cancer screening, in order to inform core elements of a strategy that would be acceptable across multiple under screened groups.	Focus group discussions	Immigrant women, aged 35–69 years (*n* = 11)	7					
C. Remschmidt (2014)	Germany	To investigate whether a social media site like Facebook is an appropriate tool to assess knowledge, attitude and uptake related to HPV vaccination in young women in Germany.	Questionnaire	Women, aged 18–25 (*n* = 1161)	5					
J. Salad (2015)	The Netherlands	To explore the perceptions of Somali women living in the Netherlands regarding measures to prevent cervical cancer.	One-on-one interviews and focus group discussions	Somalian women, aged 18–65 (*n* = 20)	7					
K.F. Salman (2012)	USA	To investigate the participation status in breast and CC screening of a group of American immigrant Arab Muslim women (AMW).	Questionnaire	Arab muslim women (*n* = 50)	6					
V. Senol (2012)	Turkey	To determine the level of knowledge and behavior of married women over 18 years regarding cervical cancer in the city of Kayseri, Turkey.	Questionnaire	Married women, 18+ (*n* = 1000)	5					
N.A.E. Shahbari (2021)	Israel	To identify and compare variables associated with mothers’ uptake of two vaccinations, influenza and HPV, among different subgroups in Arab and Jewish society in Israel.	Questionnaire	Mothers with: 1. A child in second or third grade 2. A child in the eighth grade (*n* = 693)	7					
C.R. Tatari (2021)	Denmark	To explore ethnic minority women’s own ideas and preferences for a cancer screening intervention and identify their attitudes to different strategies.	Focus group discussions	Non-western women (*n* = 37)	7					
V.N. Thomas (2005)	UK	To describe some of the factors that act as barriers to effective uptake of breast and cervical cancer screening services among black minority ethnic (BME) groups living in Brent and Harrow in the UK.	Focus group discussions	African Caribbean, African, Gujarati, Pakistani, Greek and Arabic women (*n* = 85) and men (*n* = 50)	6					
G. Turan (2021)	Turkey	To evaluate the knowledge, attitude and behaviors of people about HPV infection and the HPV vaccine.	Questionnaire	Men and women between 18–65 (*n* = 836)	5					
M. Vahabi (2016)	Canada	To explore Muslim immigrant women’s views on cervical cancer screening and the acceptability of HPV self-sampling.	Questionnaire and focus group discussions	Muslim women (*n* = 30)	7					
K.E. Wijayanti (2021)	Indonesia	To examine what factors contribute to parents’ decisions to allow or not allow their daughters to receive the HPV vaccine in Jakarta, Indonesia.	Questionnaire	Parents (*n* = 423)	5					
L.A. Wilson (2021)	Canada	To understand newcomers’ knowledge, attitudes, and beliefs (KAB) on HPV and HPV vaccination and their role in HPV vaccine acceptance.	Questionnaire	Young adults and caregivers (*n* = 50)	7					
L.P. Wong (2022)	Malaysia	To investigate HPV vaccination intention among adult married women aged 27 to 45 years and its associated factors, and their spouse/partner’s influence on HPV vaccination decision-making.	Questionnaire	Females, aged 27–45 (*n* = 794)	7					
E. Yanikkerem (2013)	Turkey	To identify knowledge about cervical cancer (CC) and Pap test (PT) and the barriers why women do not have Pap test done.	Questionnaire	Women (*n* = 1,036)	7					
C. Yeo (2018)	Singapore	To understand factors that influence women’s decisions to go for Pap smears.	Questionnaire	Postnatal women, 21+ (*n* = 268)	5					
M. Yildiz (2023)	Turkey	To investigate the relationship between individuals’ knowledge, beliefs, and vaccination status regarding human papillomavirus.	Questionnaire	Individuals from Turkey (*n* = 433)	5					
L. Zeraiq (2015)	Denmark	To explore attitudes and knowledge towards HPV vaccination among Arab mothers and their daughters.	Focus group discussions	Women (*n* = 23) and daughters (*n* = 13)	6					

### Thematic analysis

To answer the research questions, a thematic analysis was conducted to identify recurring themes in the selected literature. Based on the research questions, the following themes were extracted from literature: ‘information resources’, ‘knowledge and perceptions’, ‘misconceptions’, ‘barriers and facilitators for obtaining, processing, and understanding the information’, ‘norms and beliefs’, ‘cultural aspects’, and ‘information needs and preferences’. Table 3 shows the quantitative synthesis of these themes from the selected literature.

**Table 3:** Quantitative synthesis of recurring themes in the selected literature

Themes	Mentioned in number of studies (%)
Information resources	*N* = 31 (36%)
Knowledge and perceptions	*N* = 40 (46%)
Misconceptions	*N* = 36 (41%)
Barriers	*N* = 38 (44%)
Facilitators	*N* = 33 (38%)
Norms and beliefs	*N* = 41 (47%)
Cultural aspects	*N* = 41 (47%)
Needs and preferences	*N* = 30 (34%)

The extracted data within these themes were used to answer the research questions of this study. We used the terms ‘multiple ’ and ‘many ’ to refer to up to 10 and more than 10 articles, respectively.

*What are the knowledge and beliefs among immigrant women regarding CC, CC screening, and HPV vaccination?*

### Current sources of information

Most studies have indicated friends and family [25–36], healthcare professionals [26, 27, 29–32, 35, 37–48], and the internet and other media [26, 28, 29, 31–38, 40, 41, 45–53] as the main sources of information. Other articles also mentioned schools [28–30, 32, 38, 43] and community/home visits [40, 54] as common sources of information.

### Norms, beliefs, and cultural aspects

Multiple studies have indicated that religion plays a very important role in the decision-making process regarding participation in CC screening and HPV vaccination [31, 37, 47, 55–59]. A common aspect herein is fatalistic behavior. This is expressed in the idea that God will protect them and therefore that they do not consider themselves at risk for HPV infection or developing CC [10, 42, 43, 55, 58, 60–65]. Another very common reported belief was the association between cancer and death [49, 53, 64–67]. This has resulted in many women who do not want to know their CC screening test results [49, 54]. It is also taboo to talk about CC screening with others because of fear of their test results [10, 26, 38, 49, 52, 63, 65, 67–72]. In addition to fatalistic behavior and taboo surrounding cancer and death, studies have revealed other cultural norms and beliefs that play a role in obtaining, processing, and understanding information regarding CC, CC screening, and HPV vaccination. For example, the fact that they have only one partner and sexual activity is not allowed before marriage. This resulted in the belief that it is not necessary for them to be screened or vaccinated [27, 34, 47, 49, 58]. Furthermore, studies have indicated that the sociocultural gender norm for females is to place the health of family and significant others (i.e., their children) before their own [47, 67, 68, 73]. Additionally, studies have indicated that there is a sociocultural double standard for sexual behavior before marriage [47, 68, 70]. This means that women are expected not to have sexual intercourse before marriage, whereas for men, these rules are less strict. Many of the articles (*n* = 32) concluded that cultural considerations were necessary in the development of interventions addressing participation in CC screening and HPV vaccination among migrant women [10, 26, 28, 30–32, 36, 42, 47, 52–54, 58, 59, 61, 64, 65, 69, 70, 72–83]. Multiple studies have indicated that for immigrant women, the relationship between HPV and CC is not always clear [34, 44, 47, 69, 72]. However, other studies reported that the link between HPV and sexual behavior is well known among immigrant women [27, 28, 33, 39, 46, 48, 69]. Multiple studies have reported that parents indicate that their daughters might interpret receiving the HPV vaccination as parental approval for sexual activity [27, 44, 72, 77, 84, 85]. This could also be the other way around; if a daughter asks for the HPV vaccine, parents potentially assume that their daughter is sexually active [27]. Despite these cultural norms and beliefs, more promising perceptions of CC prevention have been reported. Although there are strict rules for women regarding (intimate) contact with other men, except for their husbands, they are allowed to see a male doctor. This is because taking care of one’s health is highly valued within one’s religion [10, 47, 86]. In the Koran, there is also a saying of the Prophet Muhammad that could be applied to this: ‘Tie your donkey to a strong post, and then trust Allah’, a saying which means ‘take precautions and then trust Allah’ [86].

### Misconceptions

Several studies have reported misconceptions regarding CC screening and HPV vaccination. First, there are many misconceptions regarding the disease itself. Many studies have reported misconceptions concerning the risk factors for CC, such as hygiene [34, 52, 54, 61, 84, 87, 88], nutrition [43, 65, 87], and heredity [10, 47, 65, 81, 87]. Additionally, the transmission route of the virus is not always clear [47, 52, 89]. Other studies have indicated that some individuals do not know to which forms of cancer the screening and/or HPV vaccine is related [54, 55, 72]. There are also misconceptions about the CC screening process. For example, a study in Turkey reported that women thought that a biopsy was taken during screening [86]. While in fact, this may only happen if you are referred to the gynecologist after a positive test result. Other studies have indicated the misconception that women think that CC screening would eliminate virginity [10, 40, 60, 61, 69]. A few studies reported confusion among their participants between HPV and HIV [27, 63]. Some studies have indicated that individuals believe that CC screening and HPV vaccination function as treatments for cancer rather than early detection and prevention of the disease, respectively [52, 55, 63, 90].

A misconception found regarding HPV infection was the idea that it could only affect females, and therefore the vaccine is only available for girls [48, 58]. Another misconception found in one study was the belief that HPV infection would be preventable by the use of oral contraceptives [46].

*What are the barriers and facilitators in obtaining, processing, and understanding information regarding CC, CC screening and HPV vaccination?*

Many (*n* = 21) studies have shown that language barriers contribute to low health literacy and pose a barrier for obtaining, processing, and understanding information [10, 25, 34, 38, 40, 43, 49, 53, 55, 57, 58, 61, 64, 65, 69–71, 80, 81, 85, 91]. The language barrier is also a barrier to accessing health care, understanding the letter of invitation and communication with healthcare professionals [67, 68, 82]. To overcome this, multiple studies on CC screening indicated that the use of audiovisual materials would be helpful [26, 53, 82, 92]. Notably, limited digital skills might hinder the use of audiovisual materials [69]. Another barrier is the lack of trust in healthcare professionals and/or the healthcare system [45, 49, 64, 70, 72, 86]. However, a study in Singapore identified an inherent trust in the government as a facilitator for women to seek and process information regarding HPV vaccination [36]. Therefore, other studies on increasing CC screening participation and HPV vaccination uptake have recommended investing in rebuilding trust [38, 57, 72, 91]. To overcome language barriers, multiple studies have suggested the use of translation services. For example, the use of a dictionary, Google Translate or an interpreter during a doctor’s appointments [10, 68, 71, 72, 82]. In fact, husbands often translate and make decisions for CC screening and HPV vaccination on behalf of their wives and children, which may lead to incomplete and biased information transfer [58, 63, 68, 79]. According to several studies, information provided by someone with a similar cultural and/or religious background could work as a facilitator for obtaining, processing, and understanding the information. Additionally, encouragement by other women or family was described to inform women’s decision-making [25, 36, 38, 47, 53, 72, 85]. Indeed, women prefer personal support when considering this sensitive topic [53, 93]. One study mentioned the use of home visits to lower the barrier for asking questions regarding CC, CC screening and HPV vaccination [41]. Furthermore, five studies specifically concluded on using multiple forms as information materials and doing this both online and offline [44, 53, 54, 82, 92]. For example, by the use of audiovisual information as well as by organizing informational meetings.

We can use the socio-ecological model to subdivide these barriers and facilitators to individual, social, and system levels [94]. This results in the following overview presented in Table 4.

**Table 4:** Overview of barriers and facilitators in socio-ecological mode [94]

Individual level	Interpersonal level	Community level	System level
Barriers: • Language barrier • Low health literacy • Limited digital skills	Barriers: • Husbands/children functioning as translators and/or decision-makers leading to incomplete and biased information transfer	Barriers: • Lack of trust in healthcare professionals	Barriers: • Lack of accessible and cultural sensitive information • Lack of trust in healthcare system
Facilitators: • Translation services (dictionary, google translate, interpreter)	Facilitators: • Encouragement by other women or family	Facilitators: • Information provided by someone with a similar cultural and/or religious background • Providing personal support (e.g. by providing home visits)	Facilitators: • Audio-visual information • Rebuilding trust • Multi-component educational materials

*What are the information needs and preferences of immigrant women regarding CC, CC screening and HPV vaccination?*

A number of articles identified a lack of knowledge and awareness as the most common barriers to obtaining, processing, and understanding the information. Therefore, one of the prominent needs these women have is receiving more information [28, 33, 36, 44, 55, 58, 68, 72, 95]. They also prefer information to be more tailored to their language, culture, and religion [44, 49, 52, 64, 72, 75, 96]. Turkish and Moroccan women in a study in the Netherlands indicated that the urgency of testing and the severity of CC were not strongly emphasized in the current information materials that are in use [69]. Additionally, there is a need for more information about the procedure steps and implications of the test findings for CC screening [43, 64]. With respect to how information is received, studies have indicated a preference for verbally transmitted health information [26, 45]. For the younger generation, this could be through social media, but the older generation is more focused on regular internet (Google, YouTube) and TV [26, 44, 45, 63]. Another way is to transmit health information verbally by organizing information meetings at mosques and community centers [10, 44, 63, 70], preferably hosted by a (known) female healthcare professional, in their own language and incorporating Islamic beliefs and values [10, 70, 81]. According to Pratt et al. (2019), this would contribute to opening the discussion about sex in relation to marriage, which is highly stigmatized within these cultures owing to religious norms and values [63]. Multiple studies have also suggested implementing this topic in school classes [33, 44, 45, 63] or integration courses [52] to increase awareness among immigrants in the early phase.

Furthermore, we compared the data between CC screening and HPV vaccination. Little to no differences were found for data concerning CC screening and HPV vaccination. All the described aspects seem to play a similar role in decision-making for both CC screening and HPV vaccination among immigrant women.

## Discussion

### Main findings

This scoping review explored how migrant women obtain, process, and understand information regarding CC, CC screening, and HPV vaccination and provided valuable insights into the barriers and facilitators in obtaining, processing, and understanding the information reported in the literature. We also provided an overview of the information needs and preferences of migrant women as reported in the literature regarding CC, CC screening, and HPV vaccination. Our results suggest notable gaps in their awareness and knowledge about, as well as a low risk perception of, CC and a negative attitude toward CC screening and HPV vaccination. We also identified several important information sources for immigrant women. The literature suggests that immigrant women tend to rely on verbal and audiovisual communication, which could be attributed to experienced language barriers and their limited health literacy. This is in line with the findings of another scoping review among immigrants and refugees in Europe on health status and healthcare [97]. This review revealed both language barriers and low health literacy as underlying causes of health inequalities between immigrants and refugees. Overall, the results of this present scoping review indicate that information regarding CC screening and HPV vaccination needs to be more tailored to the needs of immigrant women in terms of cultural, religious, and practical aspects. Information should be provided in their own language, as should the source of information, the content and the way they receive the information. Worldwide, a vast proportion of the available information sources are currently text based. This is slowly shifting toward more audiovisual information materials, but it is still not sufficiently accessible for every population group. This is mainly due to differences in digital skills and language levels. Therefore, the information materials are not tailored to the needs and preferences of immigrant women. This contributes to the existing lack of awareness and knowledge, low risk perception of CC, and negative attitudes regarding CC screening and HPV vaccination. Overall, there is a need for more information on the cultural and religious aspects of CC, CC screening, and HPV vaccination. Among the articles using a theoretical framework, the most commonly used was the HBM. This model is mainly focused on the individual level. However, this review also identified several barriers occurring at other levels of the socio-ecological model. Therefore, future research should also focus on interventions to overcome the identified barriers at interpersonal, community and system level.

### Comparison with other studies

Several cultural aspects, such as shame, fear, taboo, and partner influence have been identified in many studies as hindering some women from seeking information actively. Additionally, a scoping review also identified the religious beliefs and misconceptions towards HPV vaccination acceptance within the Organization of Islamic Cooperation (OIC) countries [98]. This study identified also similar aspects influencing women’s information seeking behavior, such as fatalism, partner influence, and the idea that HPV vaccination would be interpreted as parental approval for sexual activity. Misconceptions found in this study were the belief that the HPV vaccination contains haram substances and unnatural materials. Furthermore, there are also concerns about the vaccine causing infertility [98].

Receiving information from someone with a similar cultural and/or religious background could work as a facilitator for obtaining, processing, and understanding the information. This is because many cultural norms, values and beliefs are found to influence the decision-making process of immigrant women. For example, the impact of social norms and values related to sexuality. These factors contribute to the stigma surrounding participation in CC screening and HPV vaccination, primarily due to the association with promiscuity. These findings are further substantiated by findings from another study conducted among newly screened women in Zambia, where promiscuity was frequently cited as a risk factor for CC [99]. This association extends beyond its impact on perceptions of CC itself, since it even affects the stigma surrounding seeking information about CC screening and HPV vaccination. Individuals might be more reluctant to seek information because they fear potential reactions from their (social) environment.

Another cultural factor among immigrant women that influences the decision-making process for participation in CC screening is fatalistic behavior. This is not surprising, as previous studies have indicated an association of fatalism with being less positive about early detection [100–103]. Although this review did not identify any studies on the association of fatalism with HPV vaccination acceptance, acceptance of other types of vaccination were linked to fatalism, which poses a barrier. For example, a study on the acceptance of new variants of COVID-19 vaccines was conducted [104]. This study revealed that fatalism has a negative effect on COVID-19 vaccine acceptance and results in concerns about new variants. Although HPV vaccination was first recommended in 2006, it is still considered as a ‘new’ vaccination compared to other vaccinations in national immunization programs. Therefore, fatalism could also pose a barrier for the acceptance of HPV vaccination. However, the effect of mistrust on HPV vaccination acceptance might have a greater influence than fatalism does, since mistrust is a more common issue in HPV vaccine acceptance.

### Strengths and limitations

This scoping review provides a broader understanding of the decision-making processes of immigrant women concerning their participation in CC screening and their use of HPV vaccination for their children. It is well known that some particular study populations, such as immigrant women, are underrepresented in research [105]. This is also indicated by the fact that this scoping review included only one study among Syrian women. However, this scoping review draws attention to studies among immigrant women by providing new insights into their barriers to and facilitators of obtaining, processing and understanding information regarding CC screening and HPV vaccination. To our knowledge, this specific focus of barriers and facilitators has been lacking in the literature to date. A limitation could be that most studies included in this review were conducted in the US and Turkey. This potentially affects the generalizability of the findings to other contexts. Studies conducted in other contexts may lead to different results in terms of the knowledge, beliefs, barriers, facilitators, needs and preferences that play a role in obtaining, processing, and understanding information about CC prevention programs.

## Conclusions

Our scoping review explored information-seeking behavior and the information needs of immigrant women regarding CC, CC screening, and HPV vaccination. The identified language barriers and limited health literacy could affect immigrant women’s lack of awareness and knowledge, low risk perceptions, and negative attitudes toward CC, CC screening, and HPV vaccination. This scoping review pointed out several barriers and facilitators, at different levels of the socio-ecological model, for immigrant women in obtaining, processing, and understanding information regarding these programs. Shame, fear, taboo, and partner influence fuel the low perceived susceptibility to and severity of CC. Our scoping review suggests that there is a need for more tailored information for immigrant women. This information should contain aspects that are culturally and religiously relevant to them. For further research, we suggest investigating the specific information needs and preferences of immigrant women in more detail. These needs may vary among different immigrant populations and for specific contexts in which they are situated. To put this into practice, we conducted a semi-structured interview study among Turkish-, Moroccan-, and Syrian-Dutch women (*manuscript in preparation)*. This study aimed to gain a more in-depth understanding of their information needs and preferences regarding HPV vaccination and CC screening. Furthermore, based on the findings of this review, it is recommended to test different types and forms of information (according to these women’s needs) and outreach activities among this target population via different levels of the socio-ecological model. Currently, we are working on two intervention studies; a training for key community leaders in which they get trained to provide informational meetings within their community and short culturally sensitive videos that discuss the prominent questions within this target population including a bicultural healthcare professional and experts-by-experience from the target population. Both studies will be evaluated through quantitative and qualitative methods.

## Electronic supplementary material

Below is the link to the electronic supplementary material.

Supplementary Material 1

Supplementary Material 2

## Data Availability

Data is provided within the manuscript or supplementary information files.

## Abbreviations

CC: Cervical cancer
HPV: Human papilloma virus
WHO: World Health Organization
US: United States
ACIP: Advisory Committee on Immunization Practices
CDC: Centers for Disease Control and Prevention
SCDM: Shared clinical decision-making
PCC: Population/Concept/Context Framework
