# Supplementary material for: Barriers, facilitators, needs, and preferences in seeking information regarding cervical cancer prevention programs among Turkish, Moroccan, and Syrian immigrant women: a scoping review
Source: BMC Public Health. 2025 Apr 2;25:1242. doi: 10.1186/s12889-025-22359-2 (PMC11963620; doi:10.1186/s12889-025-22359-2)
Supplement: Supplementary file 2 — Supplementary Material 2 [file 12889_2025_22359_MOESM2_ESM.docx]

Appendix B

**Table B1: Search string Embase.com**

| Database | Embase.com | | |
| --- | --- | --- | --- |
| Concepts | **Search #** | **String** | **Hits** |
| *Prevention* | 1 | 'prevention and control'/exp/mj OR 'early diagnosis'/exp OR 'screening test'/exp/mj OR 'vaccination'/exp/mj OR 'vaccination*':ti OR 'early diagno*':ti OR 'screening*':ti | 1,168,280 |
| *Information* | 2 | 'health education'/exp/mj OR 'health behavior'/exp OR 'decision making'/exp/mj OR 'information processing'/exp OR 'community care'/exp OR 'information processing*':ti,ab OR 'decision making*':ti,ab OR 'community care*':ti,ab | 3,395,307 |
| *Target population* | 3 | 'vulnerable population'/exp OR 'susceptible population'/exp OR 'migrant'/exp/mj OR 'muslim'/exp OR 'islam'/exp OR 'arabic (language)'/exp OR 'turk (people)'/exp OR 'moroccan'/exp OR 'syrian'/exp OR 'migrant*':ti,ab OR 'muslim*':ti,ab OR 'islam*':ti,ab OR 'turk*':ti,ab OR 'moroc*':ti,ab OR 'syrian*':ti,ab | 204,266 |
|  | 4 | 'vulnerable population'/exp OR 'susceptible population'/exp OR 'migrant'/exp/mj OR 'muslim'/exp OR 'islam'/exp OR 'arabic (language)'/exp OR 'turk (people)'/exp OR 'moroccan'/exp OR 'syrian'/exp OR 'ethn*':ti OR 'migrant*':ti,ab OR 'islam*':ti,ab OR 'muslim':ti,ab OR 'turk*':ti,ab OR 'moroc*':ti,ab OR 'syria*':ti,ab | 256,814 |
| *CC and HPV* | 5 | 'uterine cervix tumor'/exp/mj OR 'wart virus'/exp/mj OR 'human papilloma virus vaccine'/exp OR 'wart virus':ti OR 'human papilloma*':ti OR hpv:ti | 134,447 |
|  | 6 | #1 AND #2 AND #3 AND #5 | 161 |
| *FINAL* | 7 | #1 AND #2 AND #4 AND #5 | 215 |

**Table B2: Database PsychInfo**

| Database | PsychInfo | | |
| --- | --- | --- | --- |
| Concepts | **Search #** | **String** | **Hits** |
| *Prevention* | 1 | ((exp Prevention/ OR Prevention.mp) OR (exp Screening/ OR Screening.mp) OR (exp Vaccination/ OR Vaccination.mp) OR (exp Early Diagnosis/) OR (vaccin* OR early diagno* OR screen*)) | 314,593 |
| *Information* | 2 | ((exp Health Education/ OR Health Education.mp) OR (exp Decision Making/ OR Decision Making.mp) OR (exp Health Behavior/ OR exp Health Information/ OR exp Community Health Care/) OR (information processing* OR decision making_ OR community care*)) | 298,392 |
| *Target population* | 3 | ((exp Immigrants/ OR Immigrants.mp) OR (exp Vulnerable Populations/ OR exp Muslims/ OR exp Islam/ OR exp Arabic language/ OR exp Turks/ OR exp Morocco/ OR exp Syrians ethnic group/) OR (migrant* OR muslim* OR islam* OR Turk* OR Moroc* OR Syria*)) | 74,331 |
|  | 4 | exp Immigrants/ or Immigrants.mp. or (exp Vulnerable Populations/ or exp Muslims/ or exp Islam/ or exp Arabic language/ or exp Turks/ or exp Morocco/ or exp Syrians ethnic group/) or (migrant* or ethn* or muslim* or islam* or arab* or Turk* or Moroc* or Syria*) | 257,436 |
| *CC and HPV* | 5 | ((exp cervical cancer/OR cervical cancer.mp) OR (exp Uterine Cervical Neoplasm/ OR exp human papillomavirus/ OR exp Human Papilloma Virus Vaccine/) OR (wart virus* OR human papilloma* OR HPV* OR cervical cancer*)) | 4144 |
|  | 6 | #1 AND #2 AND #3 AND #5 | 49 |
| *FINAL* | 7 | #1 AND #2 AND #4 AND #5 | 133 |

**Table B3: Search string CINAHL**

| Database | CINAHL |  |  |
| --- | --- | --- | --- |
| Concepts | **Search #** | **String** | **Hits** |
| *Prevention* | 1 | ( prevention and control ) OR early diagnosis OR screening test OR vaccinations OR vaccination* OR early diagno* OR screening* | 939,272 |
| *Information* | 2 | health education OR health behavior OR decision making OR information processing OR community care OR information processing* OR decision making* OR community care* | 410,615 |
| *Target population* | 3 | 'vulnerable population'/exp OR 'susceptible population'/exp OR 'migrant'/exp/mj OR 'muslim'/exp OR 'islam'/exp OR 'arabic (language)'/exp OR 'turk (people)'/exp OR 'moroccan'/exp OR 'syrian'/exp OR 'migrant*':ti,ab OR 'muslim*':ti,ab OR 'islam*':ti,ab OR 'turk*':ti,ab OR 'moroc*':ti,ab OR 'syrian*':ti,ab | 85,941 |
|  | 4 | vulnerable population OR susceptible population OR migrant* OR ( muslim or islam or islamic ) OR arabic (language) OR turk* OR moroc* OR syria* OR ethn* | 233,188 |
| *CC and HPV* | 5 | 'uterine cervix tumor'/exp/mj OR 'wart virus'/exp/mj OR 'human papilloma virus vaccine'/exp OR 'wart virus':ti OR 'human papilloma*':ti OR hpv:ti | 29,243 |
| *FINAL* | 6 | #1 AND #2 AND #3 AND #5 | 111 |
|  | 7 | #1 AND #2 AND #4 AND #5 | 111 |
